# EIF5A2 acts as a potential marker for prognosis and immunity in human cancers

**DOI:** 10.1007/s13205-025-04509-w

**Published:** 2025-09-15

**Authors:** Xuan Zhou, Yifei Sun, Qian Yang, Hong Li, Yanxia Jiang, Fenggang Xiang, Ting Liu

**Affiliations:** 1 Department of Pathology, The Affiliated Hospital of Qingdao University, 16 Jiangsu Road, Qingdao, 266000 People’s Republic of China; 2 Department of Pathology, School of Basic Medicine, Qingdao University, 308 Ningxia Road, Qingdao, 266071 People’s Republic of China; 3 Department of Pathology, Beijing Ditan Hospital, Capital Medical University, No. 8 Jing Shun East Street, Chaoyang District, Beijing, 100015 China

**Keywords:** EIF5A2, Pan-cancer, Prognosis, Immunity

## Abstract

EIF5A2, a key member of the EIF family, has not been extensively studied regarding its role and mechanism in pan-cancer. TCGA and GTEx analyses were performed to investigate the differential expression of EIF5A2 in tumors and normal tissues. cBioportal was used to investigate the gene alterations of EIF5A2 in tumors. We used Cox regression and Kaplan–Meier analyses to discuss the impact of EIF5A2 expression on prognosis. Quantitative real-time PCR (qRT-PCR) was used to detect EIF5A2 mRNA levels in 12 cases of fresh liver hepatocellular carcinoma (LIHC) and corresponding adjacent non-tumor tissues. Immunohistochemistry (IHC) was employed to evaluate the expression levels of EIF5A2 in 284 cases of LIHC as well as in adjacent non-tumorous tissues. Furthermore, the study explored the association between EIF5A2 expression and various clinicopathological parameters, along with its prognostic implications. Spearman correlation analysis was used to estimate the relationship between EIF5A2 expression and tumor mutation burden (TMB), microsatellite instability (MSI), and immunologic features. “pRRophetic”R package was utilized to obtain the sensitivity of common drugs. The Gene Set Enrichment Analysis (GSEA) was used to study the functional enrichment analysis of EIF5A2-related genes. EIF5A2 was overexpressed in most tumors using TCGA and GTEx databases. Cox regression analysis demonstrated that high EIF5A2 expression was associated with unfavorable overall survival (OS) and disease-specific survival (DSS) in head and neck squamous cell carcinoma (HNSC), brain lower grade glioma (LGG), and LIHC. The frequency of EIF5A2 gene alterations was the highest in lung squamous cell carcinoma (LUSC). EIF5A2 expression was associated with TMB, MSI, and immune cell infiltration in some tumors. We performed IHC and qRT-PCR to evaluate EIF5A2 expression in HCC and normal tissues, and found upregulation of EIF5A2 expression at the mRNA and protein levels in LIHC. There was a correlation between EIF5A2 expression and tumor size, tumor grade, and TNM stage in LIHC. Kaplan–Meier survival suggested that the overexpression EIF5A2 group had unfavorable outcomes in LIHC. EIF5A2 expression was correlated with immune cell infiltration in LIHC. The high EIF5A2 expression group was more sensitive to cisplatin, crizontinib, gemcitabine, and nilotinib in LIHC. High EIF5A2 expression was associated with several pathways, including cell cycle, proteasome, DNA replication, primary immunodeficiency and oocyte meiosis. EIF5A2 may serve as a potential prognostic marker and a latent focus for cancer immunological treatment.

## Introduction

Liver cancer is one of the five deadliest cancers globally and its incidence is increasing every year. Despite extensive research for decades, cancer continues to be a major global health problem (Prajapati et al. 2025). This is particularly prevalent in developing countries (Siegel et al. 2019; Starley et al. 2010a; Wu et al. 2019). Liver hepatocellular carcinoma (LIHC) is the most common histological subtype of liver cancer (Starley et al. 2010b). Most patients are identified with the disease in its later stages. Significant progress has been made in diagnosing diseases early through standard interventions, such as radiation, surgery, personalized strategies, and chemotherapy (Mishra et al 2023a). However, postoperative recurrence and chemotherapy resistance lead to a poor overall prognosis. Therefore, identification of therapeutic targets is crucial for molecular-targeted therapy for cancer.

Eukaryotic translation initiation factor 5A2 (EIF5A2) was first sequenced and isolated from human chromosome 3q26 in 2000 (Llovet et al. 2016; Guan et al. 2001). The EIF5A family consists of two members (EIF5A1 and EIF5A2), both of which contain a special amino acid, 8-hydroxy-2, 7, 10-triamino-capric acid (hypusine), which is vital for post-translational modification and activation of EIF5A2 (Jenkins et al. 2001). Unlike EIF5A1, which is typically expressed in normal humans, EIF5A2 has tissue-specific and cell-specific characteristics, and its mRNA is expressed only in specific tissues (such as testes, brains, and some malignant tumors) and their cells (Clement et al. 2003). Research has shown that EIF5A2 is elevated in multiple cancers, including cervical, ovarian, colorectal, gastric, liver, melanoma, lung, nasopharyngeal, gallbladder, and esophageal squamous cell carcinoma (Wang et al. 2013; Liu et al. 2017; Yang et al. 2009, 2016a, 2016b; Quanico et al. 2017; Zhu et al. 2012; Meng et al. 2015; Tang et al. 2010; Cao et al. 2017; Khosravi et al. 2014, 2016; Chen et al. 2018; Huang et al. 2016; Zheng et al. 2020; Li et al. 2014). In bladder cancer cells, EIF5A2 promotes DOX resistance, EIF5A2 causes DOX resistance through activation of the TGF-β pathway (Yang et al. 2023). EIF5A2 upregulates metastasis-associated protein 1 (MTAl) through C-MYC, thereby inducing EMT and consequently enhancing the invasion and metastasis ability of colorectal cancer cells (Zhu et al. 2011). EIF5A2 mRNA expression is significantly increased in LIHC (Yang et al. 2016b), and EIF5A2 overexpression can lead to shorter survival times (Tang et al. 2010). High expression of EIF5A2 enhances the migration and invasion ability of LIHC cells by inducing epithelial–mesenchymal transition (EMT) (Yang et al. 2016b). In this study, EIF5A2 expression, prognosis, mutation, and its relationship with tumor mutation burden (TMB), microsatellite instability (MSI), immunity, and drug sensitivity were studied from a pan-cancer perspective.

## Materials and methods

### Data preparation

We obtained the expression data of 33 types of tumors from the TCGA (version: GDC2.0) and GTEx (version 8) databases in TPM format and Batch effects were addressed via ComBat. Then the differential expression of EIF5A2 in different cancers and normal tissues was discussed using the Wilcoxon test. Meanwhile, EIF5A2 expression in human cancers and matched normal tissue from TCGA database was studied by paired *t* test.

### Correlative analysis of EIF5A2 expression with pathological stage in pan-cancer

To study whether EIF5A2 expression was linked with pathological stage in tumors, we acquired clinical features of 33 tumors from TCGA database. The Wilcoxon rank-sum test was used to investigate the correlation between EIF5A2 expression and clinicopathological characteristics.

### Prognostic value of EIF5A2 in pan-cancer

Each tumor was classified into low- and high-expression groups. This study selected overall survival (OS) and disease-specific survival (DSS) as survival indicators through univariate Cox regression, and the results were presented in the form of a forest plot. Cox regression analysis was conducted using the survival package in the R language (version 4.2.1), and a forest map and Kaplan–Meier analysis were used for visualization.

### Genes alterations, TMB, MSI analysis in pan-cancer

We used the cBioportal database (version 4.0) to analyze EIF5A2 gene alterations in pan-cancer. We used the R package maftools (version 2.8.05) to obtain tumor TMB and MSI from the literature and utilized Spearman’s correlation analysis to study the correlation between EIF5A2 expression and TMB and MSI in pan-cancer.

### Correlative analysis of EIF5A2 with immunological features in pan-cancer

The relationship between EIF5A2 expression and the immune score, stromal score, and estimate score was studied using the ESTIMATE algorithm in cancers. Moreover, the correlation between EIF5A2 and the 24 types of immune cell infiltration was assessed using ssGSEA. Immunotherapy using immune checkpoint inhibitors (ICIs) has opened a new era in the treatment of malignant tumors. Therefore, it is necessary to investigate the link between EIF5A2 and immune checkpoints in tumors.

### Immunohistochemical staining

We collected LIHC specimens and corresponding adjacent normal liver tissues from 284 patients surgically resected at the Affiliated Hospital of Qingdao University from February 2014 to October 2016. None of the patients had received radiotherapy or chemotherapy before surgery. Detailed patient demographics, inclusion/exclusion criteria, and clinicopathological data are summarized in Table 1. Patients were regularly followed up until April 29, 2020. There were 83 patients had complete follow-up data 5 years after surgery, and 31 of the patients died. The study was authorized by local institutional review boards (batch number: NO.ZYFYWZLL29132), and written informed consent was obtained from all patients before surgery. The tissues were collected, embedded, sliced at 4 μm, dewaxed with xylene, hydrated with gradient alcohol, soaked in 3%H2O2 for 20 min, and subjected to microwave repair (1 mmol/L EDTA, pH8.0). Rabbit monoclonal antibody EIF5A2 (EPR7412-50 cat.no.ab150439; 1:50, Abcam, UK) was added and incubated overnight at 4 °C. The secondary antibody (PV6000, Zhongshan Jinqiao Company) was followed by incubation for 30 min, color development by DAB, and tablet sealing. The slices were observed under a microscope. EIF5A2 expression was positive when a brownish-yellow or brown color appeared in the cytoplasm or nucleus. The evaluation criteria are described in the literature (He et al. 2011), and the scoring criteria for positive cell staining intensity were 0 (negative), 1 (weak staining), 2 (medium intensity staining), and 3 (strong staining). The scoring criteria for the percentage of positive cells were as follows: 1 point (< 25%), 2 points (25–50%), 3 points (≥ 50–75%), and 4 points (≥ 75%). EIF5A2 immunohistochemical staining was based on the percentage of the staining intensity fraction multiplied by the positive cell fraction. A score ≤ 4 was considered as low expression, and > 4 was considered as high expression.

**Table 1:** Association between EIF5A2 and clinicolpathological features in LIHC using IHC

Variables	n	Low expression	High expression	χ^2^	*P*
Type					
Liver cancer	284	107(37.68)	177(62.32)	20.48	** < 0.001**
Paracarcinoma	284	273(96.13)	11(3.87)		
Gender				1.914	0.167
Male	244	88 (36.07)	156 (63.93)		
Female	40	19 (47.50)	21 (52.50)		
Age (years)				3.102	0.078
< 57	127	55 (43.31)	72 (56.69)		
≥ 57	157	52 (33.12)	105 (66.88)		
AFP (ng/ml)				0.017	0.896
≤ 20	126	48 (38.10)	78 (61.90)		
> 20	158	59 (37.34)	99 (62.66)		
Tumor size (cm)				16.280	** < 0.001**
< 5	178	83 (46.07)	95 (53.93)		
≥ 5	106	24 (24.53)	82 (75.47)		
Vascular invasion				1.580	0.209
No	159	65 (40.88)	94 (59.12)		
Yes	125	42 (33.60)	83 (66.40)		
Tumor differentiation				5.523	**0.019**
Well—moderate	166	72 (43.37)	94 (56.63)		
Poor	118	35 (29.66)	83 (70.34)		
TNM stage				4.663	**0.031**
I–II	257	102 (29.96)	155 (70.04)		
III–IV	27	5 (7.41)	22 (92.59)		

### Quantitative real-time PCR (qRT-PCR)

Twelve pairs of fresh LIHC and non-tumorous tissues were randomly collected from patients during surgical resection and stored at − 80 °C for qRT-PCR analysis. Total RNA was extracted using TRIzol, and its purity and concentration were measured using a UV spectrophotometer. Real-time fluorescent PCR was performed after reverse transcription to obtain cDNA. Three duplicate wells were set for each sample, and the reaction conditions were as follows: pre-denaturation at 95 °C for 30 s; denaturation at 95 °C for 5 s, annealing at 60 °C for 30 s, and extension at 72 °C for 30 s; a total of 40 cycles. Wilcoxon rank sum test was applied to study the differential expression between LIHC and normal tissues.

The following primers were used in this study:

EIF5A2: 5’-TATGCAGTGCTCGGCCTTG-3’.

Reverse: 5’-TTGGAACATCCATGTTGTGAGTAGA-3’.

GAPDH: 5’-GCACCGTCAAGGCTGAGAAC-3’.

Reverse: 5’-TGGTGAAGACGCCAGTGGA-3’.

### Relationship between EIF5A2 and drug sensitivity in LIHC

To explore the application of EIF5A2 in clinical therapy, we used “pRRophetic” R package to obtain the sensitivity of common drugs. The half-inhibitory concentration (IC50) is an important indicator of the efficacy of drug therapy.

### Functional enrichment analysis of EIF5A2-related genes in LIHC

The GSEA was used to study the functional enrichment analysis of EIF5A2-related genes. Significance was defined with thresholds of *P* < 0.05 and FDR < 0.25.

### Statistical analysis

SPSS23.0 software was utilized to study the experimental data, and the difference in EIF5A2 expression between the two groups was explored by paired *t* test and Wilcoxon rank sum test. The relationship between EIF5A2 and clinicopathological parameters was examined using χ^2^ tests. Survival was assessed using the Kaplan–Meier survival curve and log-rank tests. Spearman correlative analysis was applied to discuss the relationship of EIF5A2 with immunity. Statistical significance was set at *P* < 0.05.

## Results

### Expression of EIF5A2 in 33 types of tumors

**Fig. 1:**
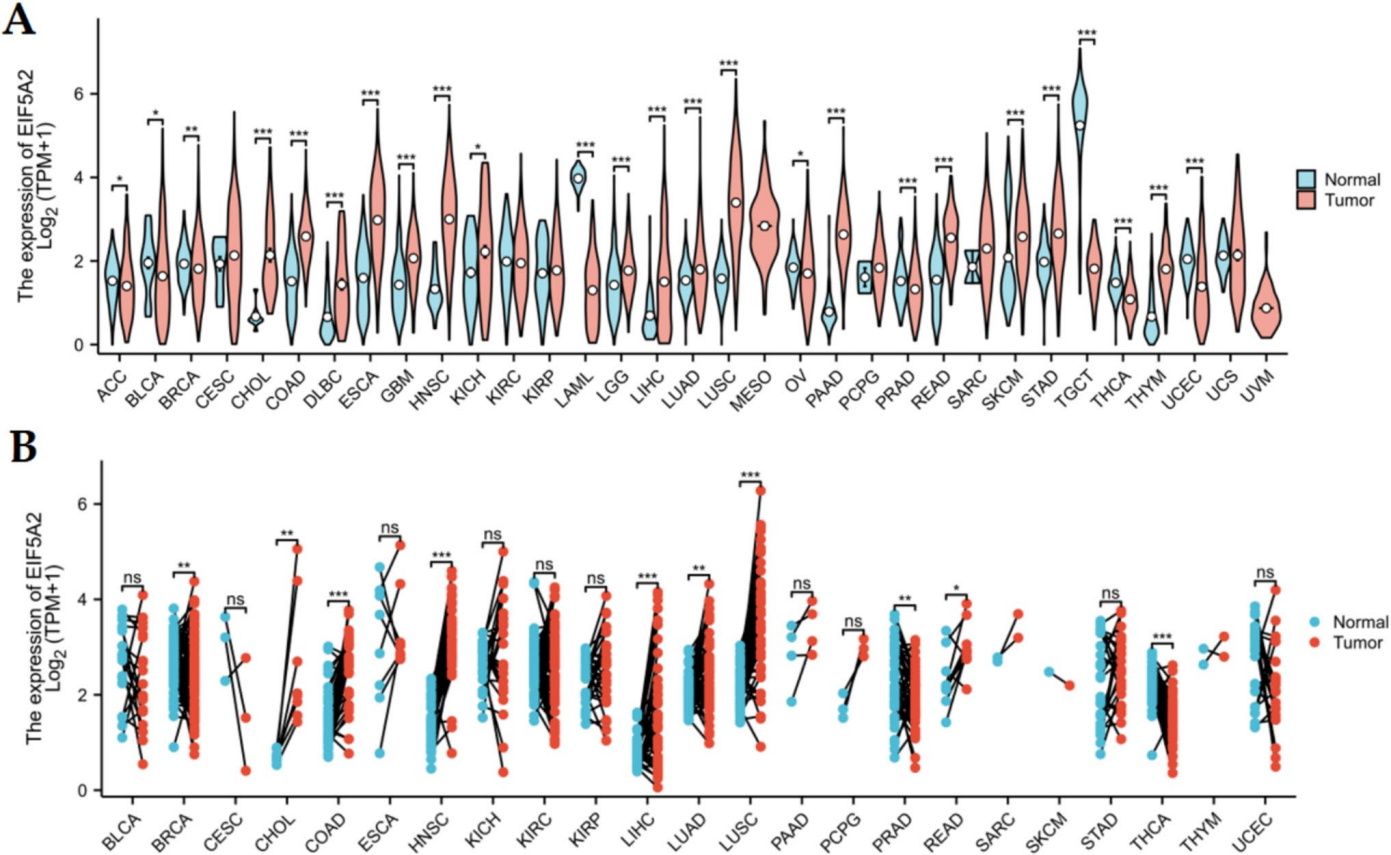
Differential expression of EIF5A2 in pan-cancer and normal tissues. **A** Differential expression of EIF5A2 in pan-cancer and normal tissues in TCGA and GTEx database. **B** Differential expression of EIF5A2 in human cancers and matched normal tissues by paired *t* test from TCGA

Using TCGA and GTEx databases, we found that EIF5A2 expression was elevated in 16 tumors relative to normal tissues, including CHOL, COAD, DLBC, ESCA, GBM, HNSC, KICH, LGG, LIHC, LUAD, LUSC, PAAD, READ, SKCM, STAD, and THYM, but decreased in 9 tumors, including ACC, BLCA, BRCA, LAML, OV, PRAD, TGCT, THCA, and UCEC (Fig. 1A). In addition, match paired analysis showed that EIF5A2 was overexpressed in CHOL, COAD, HNSC, LIHC, LUAD, LUSC, and READ and low expression in BRCA, PRAD, and THCA (Fig. 1B).

### EIF5A2 expression was related to clinicopathologic stage in some tumors

**Fig. 2:**
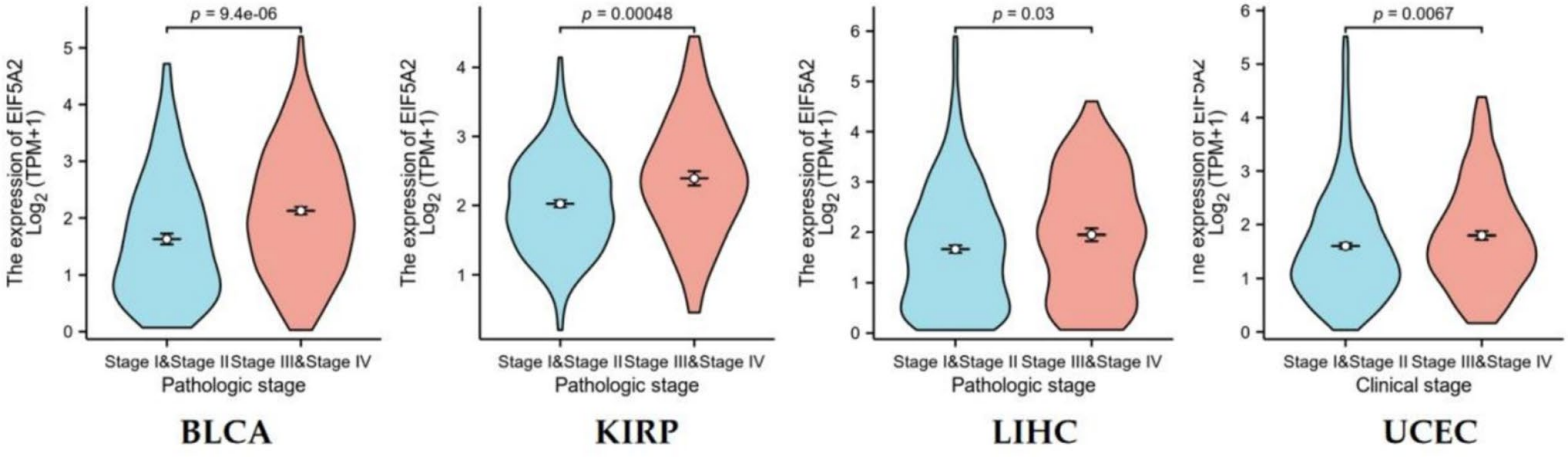
Correlation of EIF5A2 expression with clinicopathologic stage in BLCA, KIRP, LIHC, and UCEC

We selected pathological stage as a clinical index to explore its correlation with EFI5A2 expression. The results suggested that EIF5A2 expression increased in the advanced stages of BLCA, KIRP, LIHC, and UCEC (Fig. 2).

### EIF5A2 overexpression suggests an unfavorable prognosis in several tumors

**Fig. 3:**
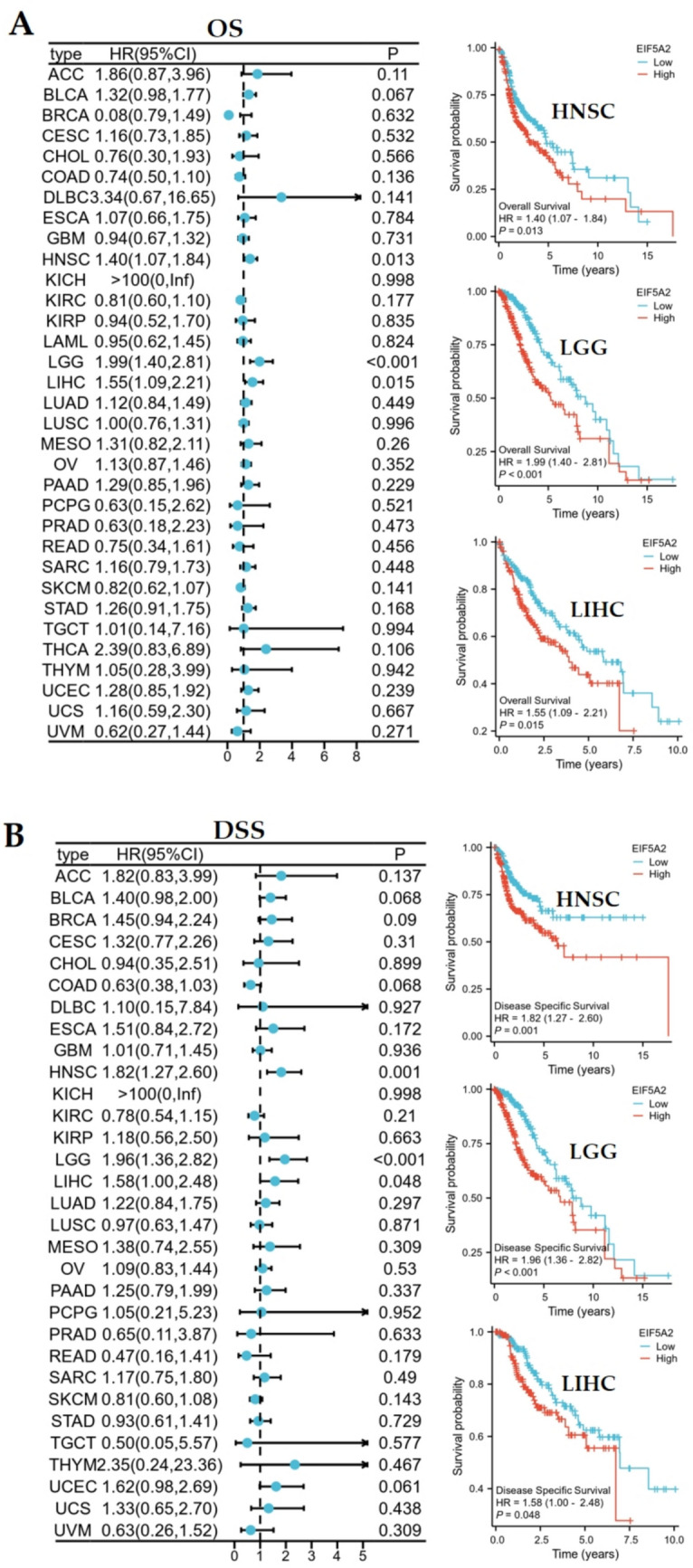
Survival analysis. **A** Univariate Cox regression and Kaplan–Meier survival curve of EIF5A2 for OS in pan-cancer. **B** Univariate Cox regression and Kaplan–Meier survival curve of EIF5A2 for DSS in pan-cancer

Overexpression of EIF5A2 is a risk factor for OS in HNSC (HR = 1.4, 95%CI 1.07–1.84, *P* = 0.013), LGG (HR = 1.99, 95%CI 1.40–2.81, *P* < 0.01), and LIHC (HR = 1.55, 95%CI 1.09–2.21, *P* = 0.015). Kaplan–Meier survival curves showed that patients with high EIF5A2 expression had shorter OSin HNSC, LGG, and LIHC (Fig. 3A). DSS analysis demonstrated that high EIF5A2 expression was a hazard index for HNSC (HR = 1.82, 95%CI 1.27–2.60, *P* = 0.001), LGG (HR = 1.96, 95%CI 1.36–2.82, *P* < 0.001), and LIHC (HR = 1.58, 95%CI 1.00–2.48, *P* = 0.048), and Kaplan–Meier analysis also revealed that overexpression of EIF5A2 had poor prognosis for these 3 tumors (Fig. 3B).

### EIF5A2 genetic alteration, TMB, and MSI analysis in various tumors

**Fig. 4:**
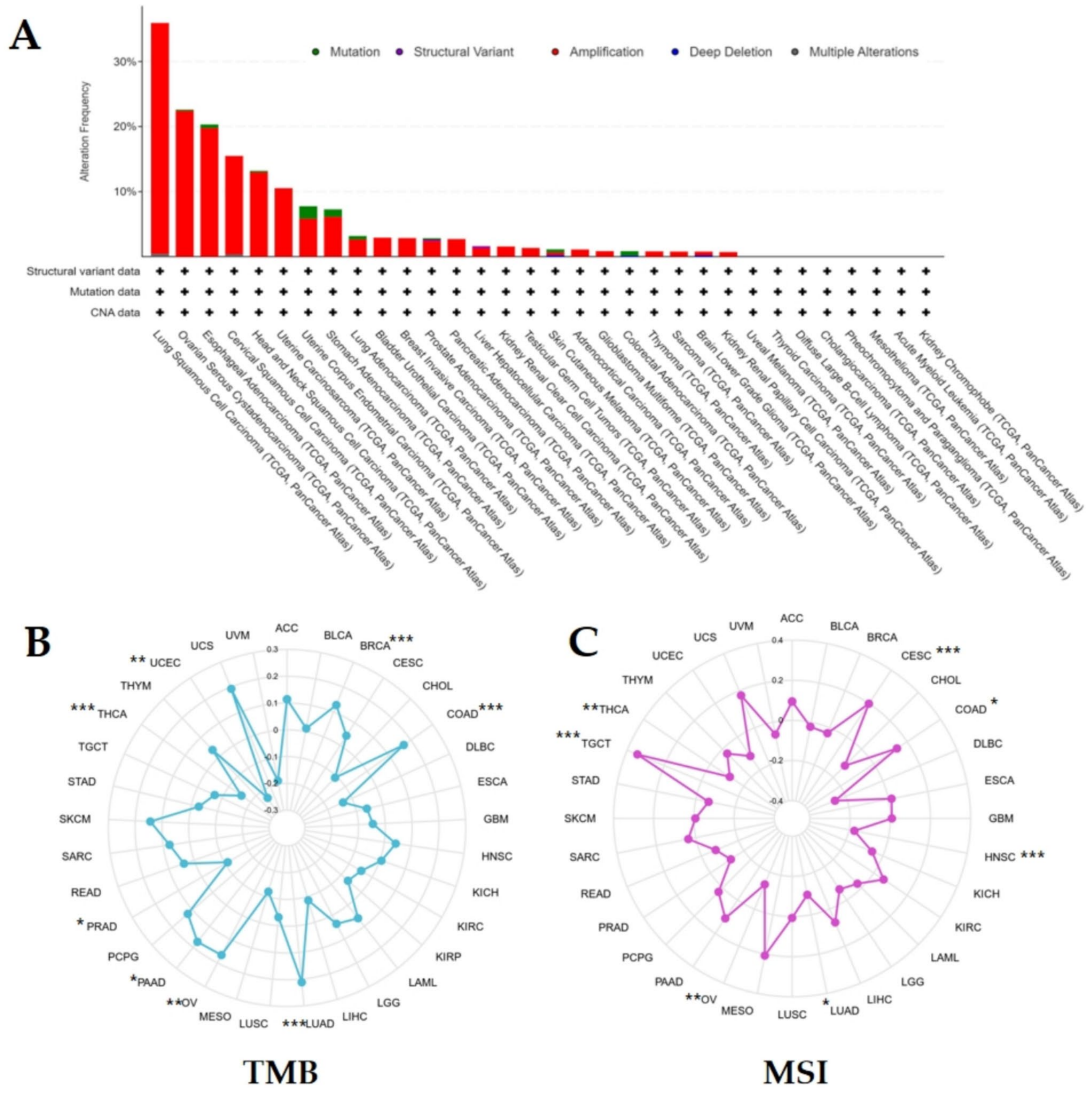
Correlative analysis. **A** Genetic alteration of EIF5A2 expression in pan-cancer. **B** Relationship between EIF5A2 expression and TMB in pan-cancer. **C** Relationship between EIF5A2 expression and MSI in pan-cancer

The cBioportal database showed that EIF5A2 was mainly present in various tumors in the form of amplification. LUSC showed the highest frequency of genetic changes, followed by OV and ESCA. No genetic alterations in EIF5A2 were observed in UVM, THCA, DLBC, CHOL, PCPG, MESO, LAML, and KICH (Fig. 4A). In addition, EIF5A2 expression was positively correlated with TMB in BRCA, COAD, LUAD, OV, and PAAD and negatively correlated with TMB in PRAD, THCA, and UCEC (Fig. 4B). MSI analysis demonstrated that EIF5A2 was positively correlated with MSI in CESC, COAD, and TGCT and negatively correlated with MSI in HNSC, LUAD, OV, and THCA (Fig. 4C).

### The relationship of EIF5A2 expression with immunological features in pan-cancer

**Fig. 5:**
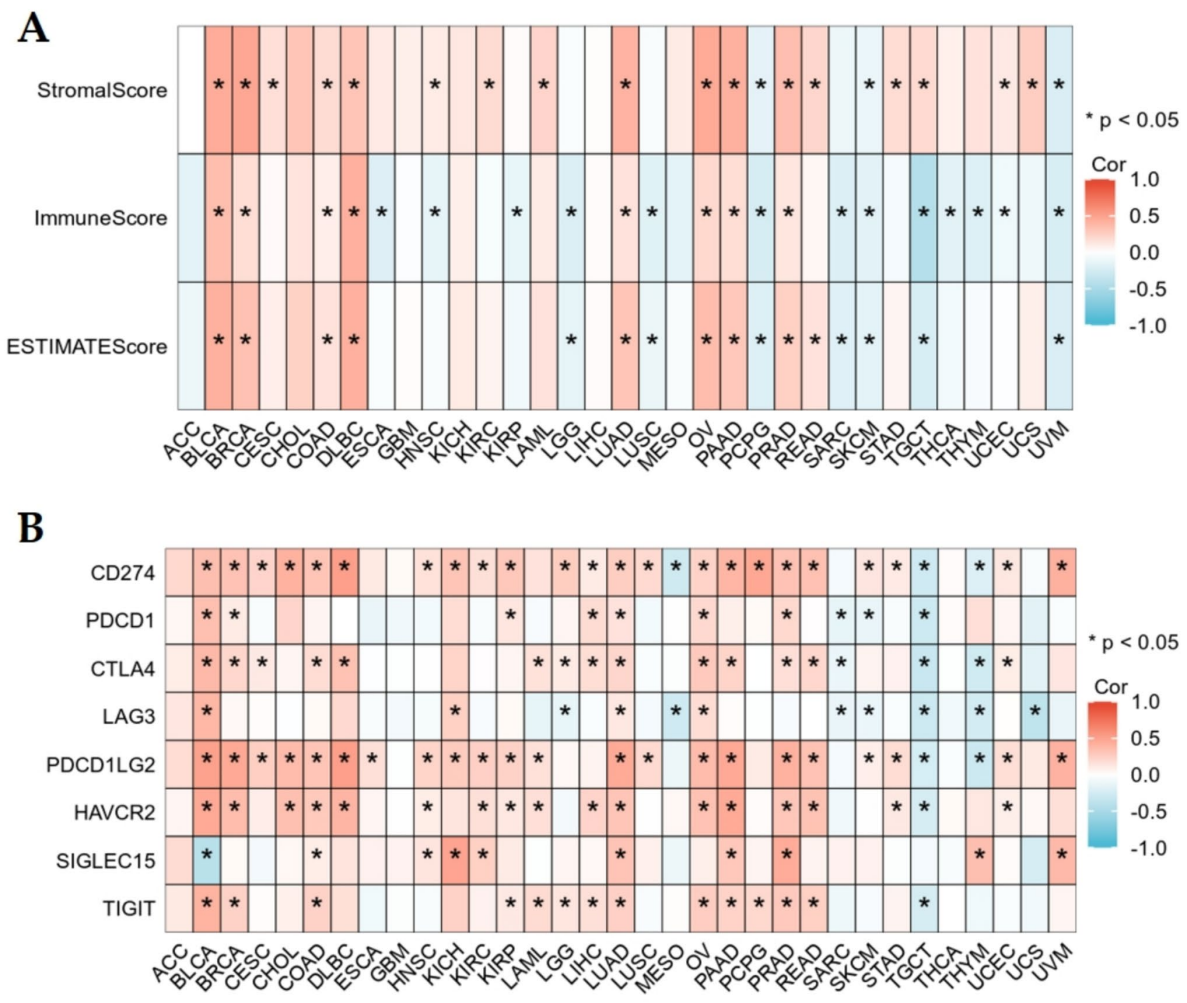
Correlation of EIF5A2 with stromal score, immune score, estimate score (**A**), and immune checkpoints (**B**)

ESTIMATE analysis demonstrated a positive association between EIF5A2 expression and stromal score, immune score, and estimate score in BLCA, BRCA, COAD, DLBC, LUAD, OV, PAAD, and PRAD, and a negative association in SKCM and UVM. EIF5A2 expression was not correlated with three scores in ACC, CHOL, GBM, KICH, LIHC, and MESO (Fig. 5A). We examined the relationship between EIF5A2 and immune checkpoints using Spearman analysis and found that EIF5A2 was correlated with CD274, PDCD1LG2, and HAVCR2 in the majority of cancers and PDCD1, CTLA4, LAG3, SIGLEC15, and TIGIT in some tumors. EIF5A2 was positively linked to CD274, PDCD1, CTLA4, HAVCR2, and TIGIT in LIHC (Fig. 5B).

### Expression of EIF5A2, prognosis and relationship with clinicopathology in LIHC by immunohistochemistry (IHC)

**Fig. 6:**
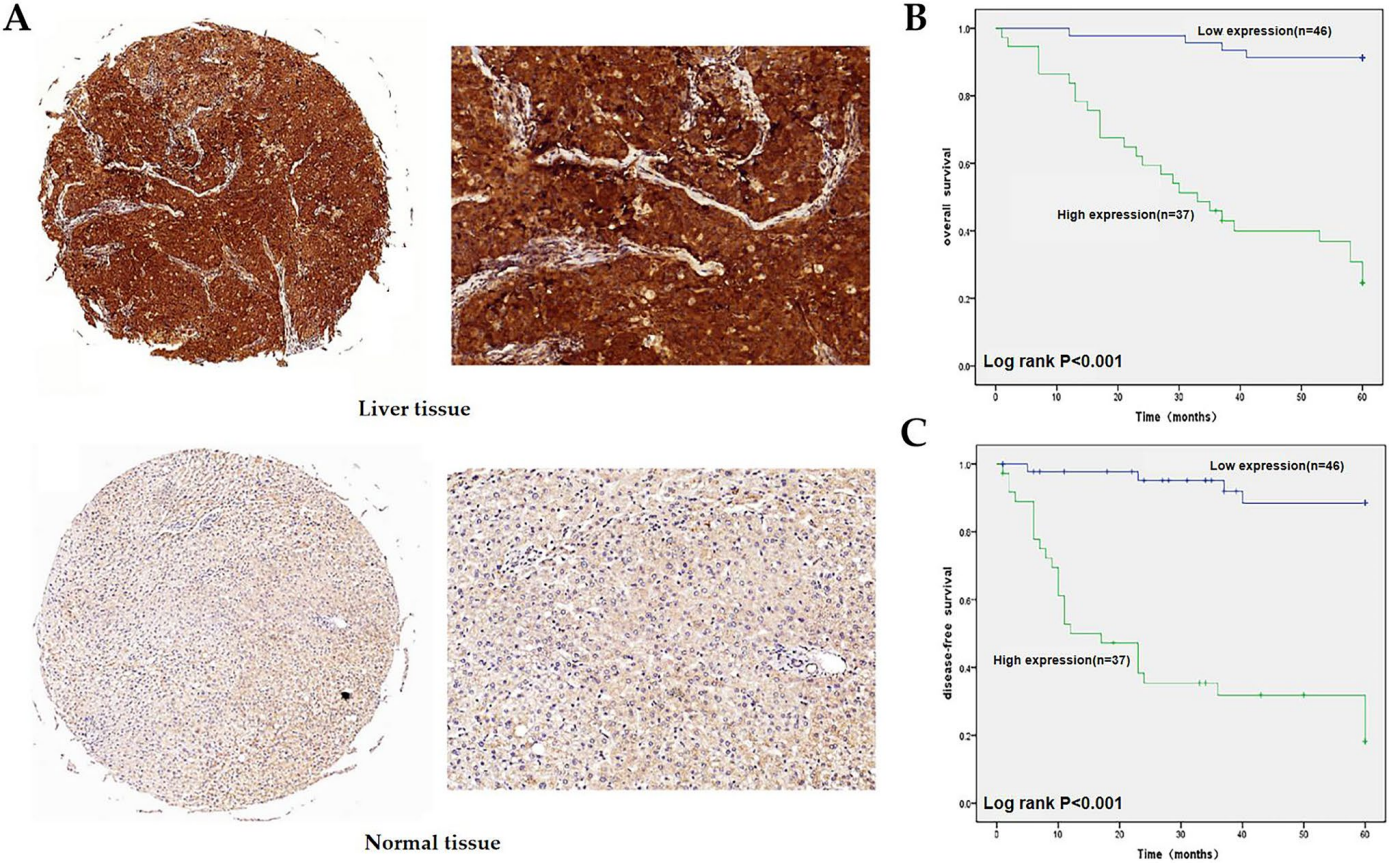
EIF5A2 expression and prognosis in LIHC. **A** IHC analysis showed upregulation of EIF5A2 in LIHC. **B, C** Overexpression of EIF5A2 had poor OS and DFS

The IHC results indicated that EIF5A2 was expressed in both the cytoplasm and nucleus, and its expression was higher than that in normal liver tissue (Fig. 6A). In addition, EIF5A2 expression was associated with tumor size, tumor grade, and TNM stage in LIHC (*P* < 0.05), but not with sex, age, AFP level, and vascular invasion (Table 1). The median OS and DFS for the high-expression group were 34.7 months and 20.3 months, respectively, whereas the medians for the low-expression group were 61.7 months and 60.2 months. The group with high EIF5A2 expression had poor OS according to Kaplan–Meier survival analysis (Fig. 6B). The group with low EIF5A2 expression had better DFS than the group with high EIF5A2 expression (Fig. 6C).

### EIF5A2 was up-regulated at level of mRNA in LIHC

**Fig. 7:**
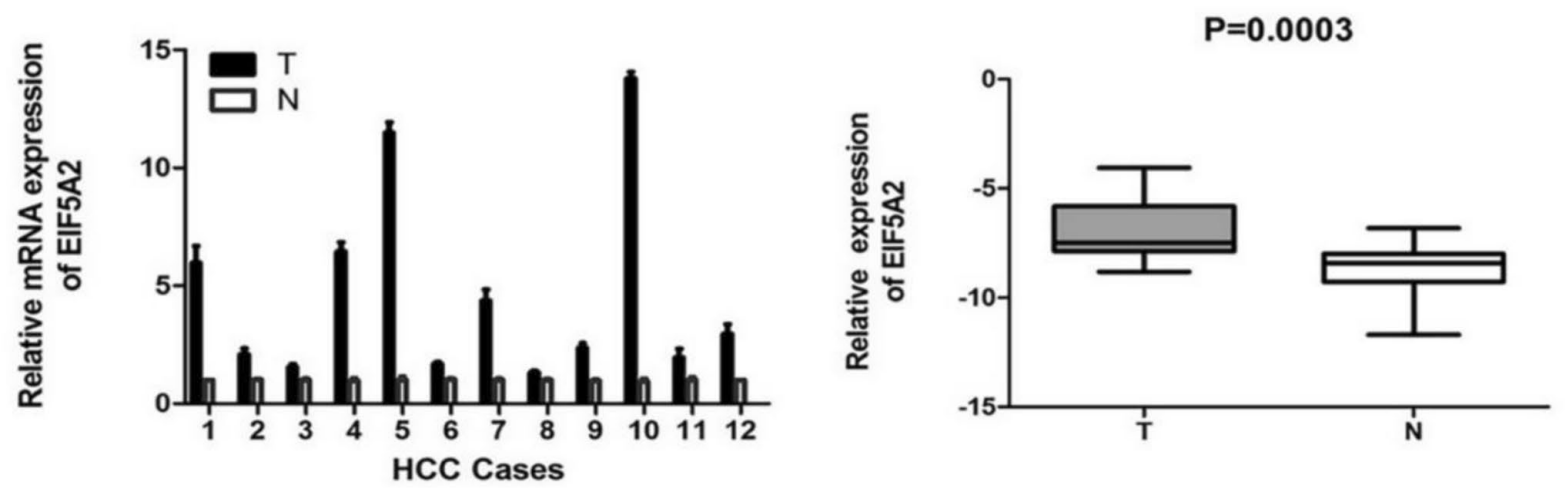
EIF5A2 expression was detected by qRT-PCR in 12 cases of LIHC and the adjacent normal tissue

EIF5A2 mRNA expression in 12 LIHC and normal tissues was observed by qRT-PCR. The findings suggested that EIF5A2 mRNA expression was significantly higher in LIHC tissues than in normal tissues (*t* = 5.305, *P* = 0.0003) (Fig. 7).

### The relationship of EIF5A2 expression with immunity in LIHC

**Fig. 8:**
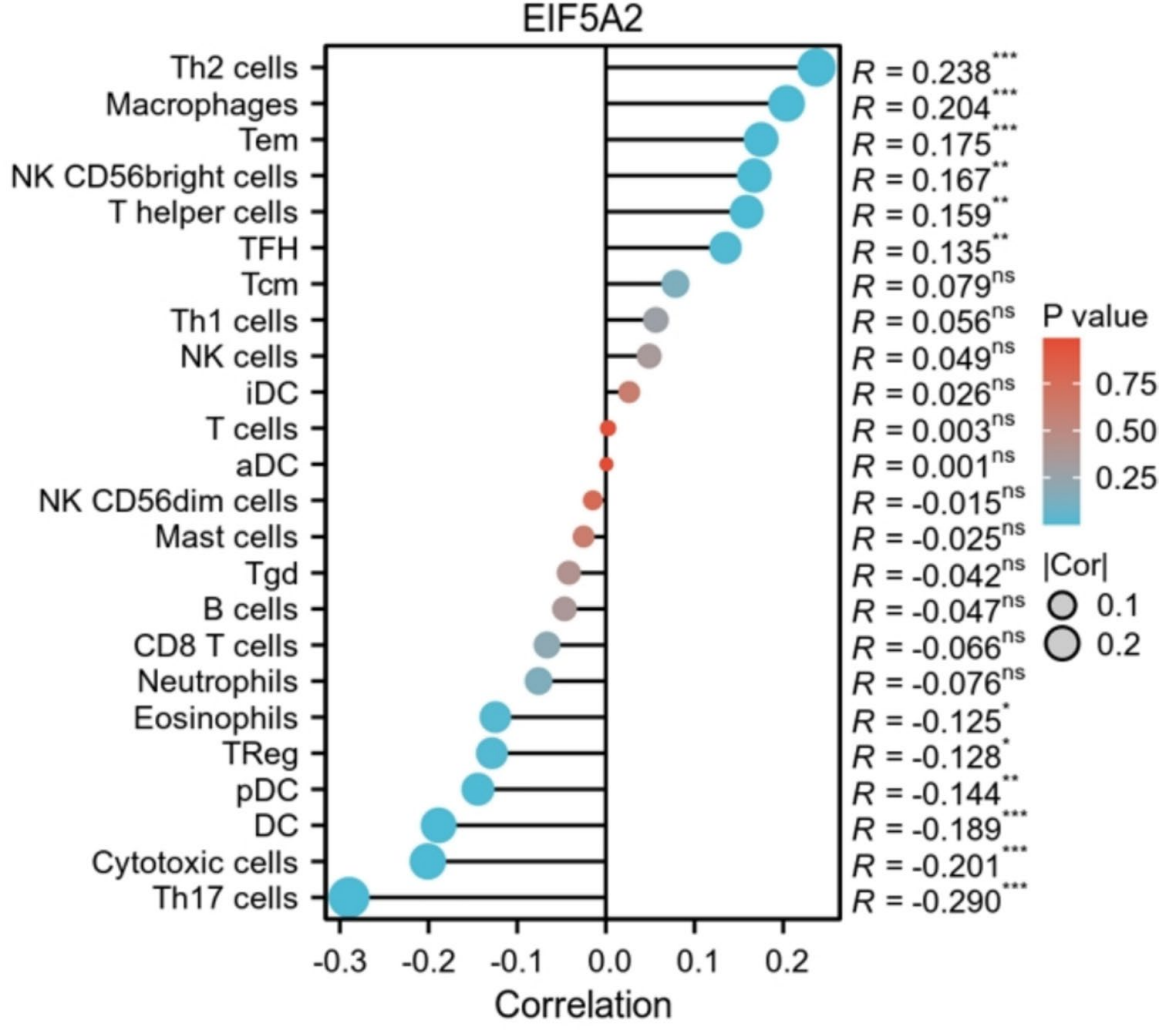
Relationship of EIF5A2 with immune cell infiltration using ssGSEA in LIHC

ssGSEA analysis demonstrated that EIF5A2 expression was positively linked with Th2 cells, macrophages, Tem, NK CD56 bright cells, T helper cells, and TFH, and negatively associated with Th17 cells, cytotoxic cells, DC, pDC, Tregs, and eosinophils (Fig. 8).

### Relationship between EIF5A2 and drug sensitivity in LIHC

**Fig. 9:**
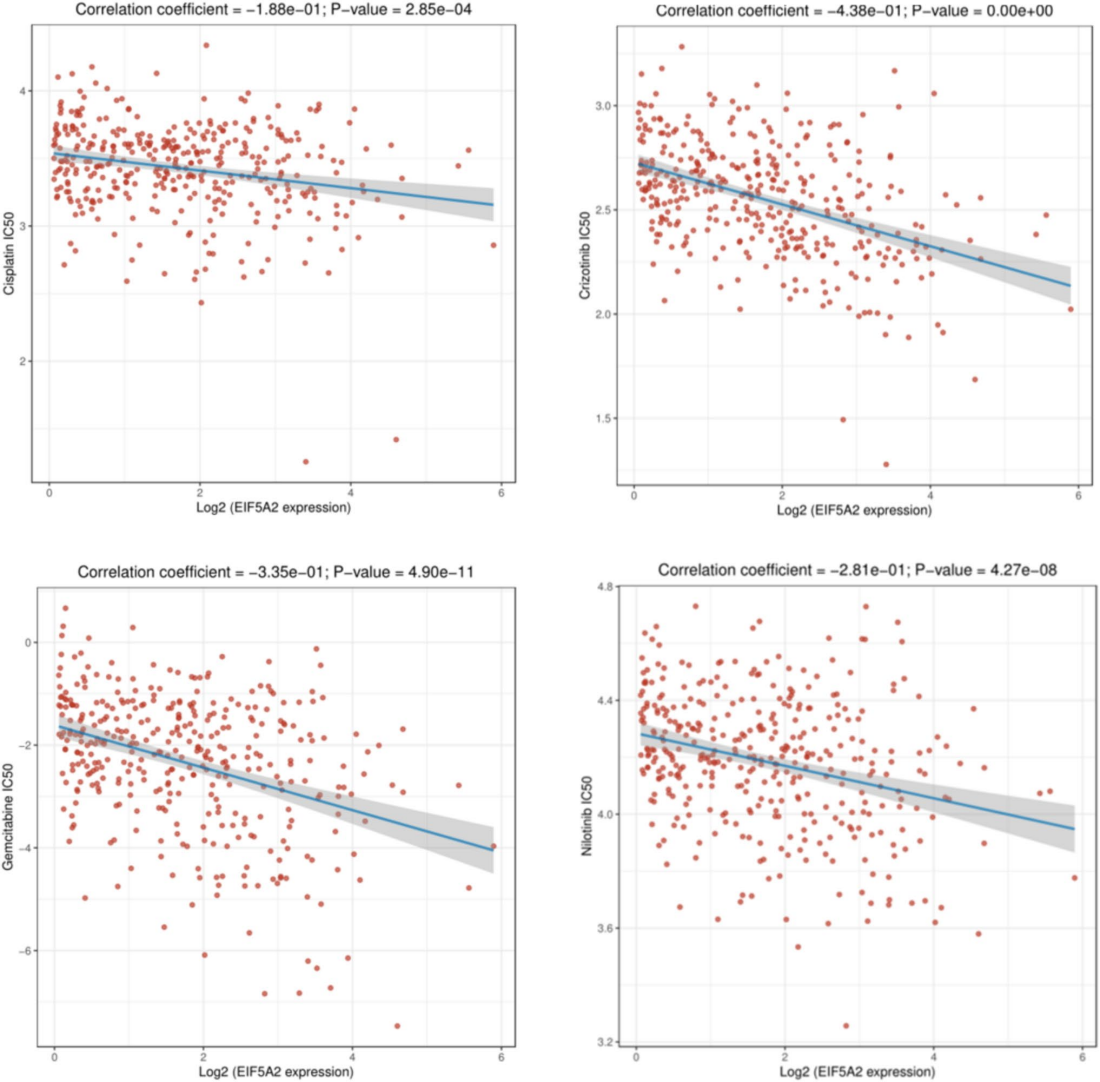
Correlation between EIF5A2 expression and drug sensitivity in LIHC

We used IC50 to assess chemotherapeutic drugs for LIHC. The lower the IC50 value of a drug, the more sensitive the patient is to the drug. The results showed that EIF5A2 expression had a negative correlation with the IC50 values of Cisplatin, Crizontinib, Gemcitabine, Nilotinib , suggesting that patients with high EIF5A2 expression were more susceptible to these drugs (Fig. 9).

### Function enrichment analysis of EIF5A2-related genes in LIHC and other tumors

**Fig. 10:**
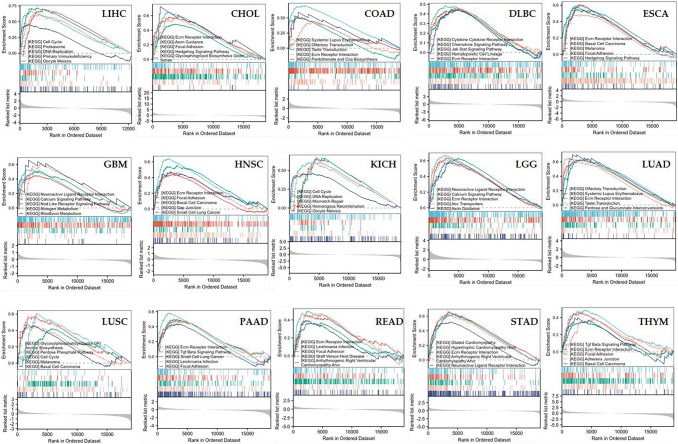
GSEA enrichment function of EIF5A2-related genes in LIHC and other tumors with EIF5A2 overexpression

To elucidate the bioprocesses involved in both groups, we utilized the KEGG set of GSEA for enrichment analysis. In LIHC, GSEA suggested that EIF5A2-related genes were involved in cell cycle, proteasome, DNA replication, primary immunodeficiency and oocyte meiosis. In most tumors, EIF5A2 was mostly enriched in ECM receptor interaction (Fig. 10).

## Discussion

Cancer is a severe disease that results in the loss of millions of lives each year (Mishra and et al 2023b; Divya Mishra et al. 2023), and according to the Global Cancer Observatory (GLOBOCAN) of the World Health Organization, a total of 18.08 million new cancer cases have been diagnosed worldwide in 2018, with liver cancer ranking fourth among men (600,000 cases) (Mattiuzzi and Lippi 2019). In China, the incidence rate of liver cancer ranks second among men and fifth among women (Feng et al. 2019). Since the early symptoms of LIHC patients are not typical, about half of the patients are in the middle and late periods when they visit a doctor, the recurrence and metastasis rates are high after surgery, and the prognosis is poor (Zeng et al. 2018). Less than 15% of patients with early liver cancer have the opportunity to undergo liver resection, while advanced patients have to choose transcatheter arterial chemoembolization (TACE) and the oral chemotherapy drug sorafenib. However, it has limited curative effects and obvious drug resistance (Anwanwan et al. 2020; El-Serag et al. 2008). Molecular targeted therapy uses drugs to prevent cancerous cell growth, and the emergence of this therapy is a milestone (El-Serag et al. 2008). At present, there is a pressing need to elucidate the pathogenic mechanisms of LIHC and identify the available targeting markers.

The EIF5A family consists of two members: EIF5A1 and EIF5A2. Recent evidence suggests that EIF5A1 is a shuttle protein present in the nucleus, cytoplasm, and mitochondria (Jao and Yu Chen 2002; Wu et al. 2020). Like the EIF5A1 protein, EIF5A2 protein is also a shuttle protein, and knockdown of expotin-4 led to nuclear accumulation of EIF5A2 protein, indicating that expotin-4 mediated EIF5A2 protein export from the nucleus (Zender et al. 2008). As an oncogene, EIF5A2 can promote the growth, invasion, and metastasis of tumor cells (Tang et al. 2010). Studies have demonstrated that EIF5A2 expression is elevated in ovarian cancer, cervical cancer, gastric cancer, lung cancer, liver cancer, and other cancers (Quanico et al. 2017; Yang et al. 2009, 2016b, 2023; Zhu et al. 2011, 2012; Meng et al. 2015; Tang et al. 2010; Cao et al. 2017; Khosravi et al. 2014, 2016; Chen et al. 2018; Huang et al. 2016; Zheng et al. 2020; Li et al. 2014), and plays a pivotal role in many biological processes, such as tumor formation, growth, and metastasis. Overexpression of EIF5A2 can induce EMT to promote liver cancer cell metastasis, and silencing of EIF5A2 can enhance the sensitivity to 5-fluorouracil (5-FU) in liver cancer cells by blocking the p38 MAPK and JNK/c-Jun/MMP-2 signaling pathways (Wang et al. 2014). Knocking down EIF5A2 can reverse EMT by inhibiting ROS pathway, thereby inhibiting liver cancer cells' invasion and metastasis (Yang et al. 2016b; Liu et al. 2016). Compared to normal gallbladder tissues, EIF5A2 expression is elevated in gallbladder cancer (Huang et al. 2016).

We analyzed the expression, prognosis, and mutation of EIF5A2 and its relationship with TMB and MSI in pan-cancers. EIF5A2 expression was upregulated in 16 tumors and downregulated in 9 tumors by TCGA and GTEx. In addition, we detected EIF5A2 mRNA expression levels in 12 pairs of fresh HCC and corresponding non-tumor tissues by qRT-PCR and found that EIF5A2 mRNA levels were upregulated in LIHC, indicating that EIF5A2 participates in LIHC progression. Cox regression analysis showed that highly expressed EIF5A2 was associated with poor OS and DSS in HNSC, LGG, and LIHC. We applied Kaplan–Meier survival curve analysis and found that patients with high EIF5A2 expression had poorer OS and DFS. We also conducted a correlation analysis between EIF5A2 and clinicopathological characteristics and showed that EIF5A2 expression was correlated with tumor size, differentiation degree, and TNM stage, but not with gender, age, AFP level, and vascular invasion. These findings demonstrate that EIF5A2 is involved in LIHC progression and may be a latent prognostic biomarker for LIHC.

Tumorigenesis is a complicated process that involves genetic changes. Gene alterations included amplification, mutations, deep deletions, structural variants, and multiple alterations. The cBioportal database has shown that EIF5A2 exists primarily in amplified forms in different tumors. LUSC showed the highest frequency of gene changes, followed by OV and ESCA. TMB is an index used to evaluate the frequency of gene mutations, and has become a latent marker for predicting the efficacy of cancer immunotherapy in clinical practice. Previous studies have confirmed that a high tumor mutation load is positively correlated with immune treatment effects (Jiao et al. 2019; Hellmann et al. 2018). This study elucidated that EIF5A2 expression was positively correlated with TMB in 5 tumors, and negatively correlated with TMB in 3 tumors. This indicates that the high expression of EIF5A2 in BRCA, COAD, LUAD, OV, and PAAD has shown good efficacy in immunotherapy. MSI is defined as a type of high microsatellite mutation rate caused by MMR defects in phenotypes (Baretti and Le 2018). Tumor cells are more prone to gene mutations due to MMR defects; therefore, MSI is one of the main factors in the development of tumor cells and is also a biological marker for predicting the effectiveness of immunotherapy (Lower et al. 2018). In CESC, COAD, and TGCT, EIF5A2 expression was positively correlated with MSI and negatively correlated with HNSC, LUAD, OV, and THCA, indicating that high EIF5A2 expression in CESC, COAD and TGCT could benefit from immunotherapy. Tumors exhibiting high TMB and high MSI show enhanced responsiveness to immunotherapy (Filipovic et al. 2020; Reck et al. 2019). High EIF5A2 expression may indirectly increase TMB by regulating DNA damage repair-related genes, thereby generating more new antigens. However, it may also activate immune checkpoint molecules (such as PD-L1) to evade immune surveillance.

The tumor microenvironment (TME) is defined as the internal and external environment of tumor cells during tumor occurrence and metastasis. Immunocytes play an important role in TME. The results demonstrated that EIF5A2 expression was positively associated with stromal score, immune core, and estimate score in some tumors by ESTIMATE analysis. We used ssGSEA to discuss the relationship between EIF5A2 expression and immune cells in LIHC and revealed that EIF5A2 expression was positively correlated with Th2 cells, macrophages, Tem, NK CD56 bright cells, T helper cells, and TFH, and negatively correlated with Th17 cells, cytotoxic cells, DC, pDC, Tregs, and eosinophils. This suggests that immune cell interactions with tumor cells can lead to tumor development. Along with the basis and continuous breakthrough of clinical research, immunotherapies such as immune checkpoint inhibitors (ICI) based on T cells have shown good efficacy in cancers. In this study, EIF5A2 expression was positively associated with common immune checkpoints in some tumors. In addition, we conducted a correlation analysis of the drug sensitivity of EIF5A2 in LIHC. In LIHC, Cisplatin, Crizontinib, Gemcitabine and Nilotinib may have a good therapeutic effect in patients with high EIF5A2 expression. To clarify the biological process of EIF5A2 in LIHC, we conducted GSEA functional enrichment analysis and found that the group with high EIF5A2 expression participated in the following pathways: cell cycle, proteasome, DNA replication, primary immunodeficiency and oocyte meiosis.

In this study, we examined EIF5A2 expression, prognosis, clinicopathological characteristics, and immunity from a pan-cancer perspective. However, some limitations of this study should be considered. First, we only examined the overexpression of EIF5A2 at the mRNA and protein levels in LIHC. The biological mechanism of EIF5A2 involvement in LIHC has not yet been elucidated and requires further study. Second, confounding factors such as tumor heterogeneity or stromal contamination in bulk RNA-seq were not considered. Further investigation is warranted to elucidate the pathological significance of EIF5A2 across a diverse range of tumors.

## Conclusion

Thus, EIF5A2 is a potential prognostic biomarker for cancer. Moreover, EIF5A2 expression was elevated at the mRNA and protein levels in LIHC tissues compared to that in normal tissues. Overexpression of EIF5A2 leads to unfavorable prognosis in some tumors. EIF5A2 expression correlates with immune cell infiltration and immune checkpoints in several cancers. This study sheds light on EIF5A2's role in various tumors and serves as a latent biomarker for prognosis and immunotherapy.

## Data Availability

The data are available from the corresponding author for reasonable requests.

## Abbreviations

ACC: Adrenocortical carcinoma
BLCA: Bladder urothelial carcinoma
BRCA: Breast invasive carcinoma
CESC: Cervical squamous cell carcinoma and endocervical adenocarcinoma
CHOL: Cholangio carcinoma
COAD: Colon adenocarcinoma
DLBC: Lymphoid neoplasm difuse large B-cell lymphoma
ESCA: Esophageal carcinoma
GBM: Glioblastoma multiforme
HNSC: Head and neck squamous cell carcinoma
KICH: Kidney chromophobe
KIRC: Kidney renal clear cell carcinoma
KIRP: Kidney renal papillary cell carcinoma
LAML: Acute myeloid leukemia
LGG: Brain lower grade glioma
LIHC: Liver hepatocellular carcinoma
LUAD: Lung adenocarcinoma
LUSC: Lung squamous cell carcinoma
MESO: Mesothelioma
OV: Ovarian serous cystadenocarcinoma
PAAD: Pancreatic adenocarcinoma
PCPG: Pheochromocytoma and paraganglioma
PRAD: Prostate adenocarcinoma
READ: Rectum adenocarcinoma
SARC: Sarcoma
SKCM: Skin cutaneous melanoma
STAD: Stomach adenocarcinoma
TGCT: Testicular germ cell tumors
THCA: Thyroid carcinoma
THYM: Thymoma
UCEC: Uterine corpus endometrial carcinoma
UCS: Uterine carcinosarcoma
UVM: Uveal melanoma
